# The Alzheimer's Disease Neuroimaging Initiative Neuropathology Core: An update

**DOI:** 10.1002/alz.14253

**Published:** 2024-10-01

**Authors:** Richard J. Perrin, Erin E. Franklin, Haley Bernhardt, Aime Burns, Katherine E. Schwetye, Nigel J. Cairns, Michael Baxter, Michael W. Weiner, John C. Morris, Alzheimer's Disease Neuroimaging Initiative

**Affiliations:** ^1^ Department of Pathology and Immunology Washington University School of Medicine Saint Louis Missouri USA; ^2^ Knight Alzheimer Disease Research Center Washington University School of Medicine Saint Louis Missouri USA; ^3^ Department of Neurology Washington University School of Medicine Saint Louis Missouri USA; ^4^ Living Systems Institute, Faculty of Health and Life Sciences University of Exeter Exeter Devon UK; ^5^ Department of Veterans Affairs Medical Center Center for Imaging of Neurodegenerative Diseases San Francisco California USA; ^6^ Department of Radiology and Biomedical Imaging University of California San Francisco San Francisco California USA; ^7^ Department of Medicine University of California San Francisco San Francisco California USA; ^8^ Department of Psychiatry and Behavioral Sciences University of California San Francisco San Francisco California USA; ^9^ Department of Neurology University of California San Francisco San Francisco California USA

**Keywords:** Alzheimer's Disease Neuroimaging Initiative, Alzheimer, biomarker, Lewy body, neuropathology, TAR DNA binding protein 43

## Abstract

**INTRODUCTION:**

Biomarkers for Alzheimer's disease neuropathologic change (ADNC) have been instrumental in developing effective disease‐modifying therapeutics. However, to prevent/treat dementia effectively, we require biomarkers for non‐AD neuropathologies; for this, neuropathologic examinations and annotated tissue samples are essential.

**METHODS:**

We conducted clinicopathologic correlation for the first 100 Alzheimer's Disease Neuroimaging Initiative (ADNI) Neuropathology Core (NPC) cases.

**RESULTS:**

Clinical syndromes in this cohort showed 95% sensitivity and 79% specificity for predicting high/intermediate ADNC, a 21% false positive rate, and a ∼44% false negative rate. In addition, 60% with high/intermediate ADNC harbored additional potentially dementing co‐pathologies.

**DISCUSSION:**

These results suggest that clinical presentation imperfectly predicts ADNC and that accurate prediction of high/intermediate ADNC does not exclude co‐pathology that may modify presentation, biomarkers, and therapeutic responses. Therefore, new biomarkers are needed for non‐AD neuropathologies. The ADNI NPC supports this mission with well‐characterized tissue samples (available through ADNI and the National Institute on Aging) and “gold‐standard” diagnostic information (soon to include digital histology).

**Highlights:**

- The Alzheimer's Disease Neuroimaging Initiative (ADNI) Neuropathology Core (NPC) brain donation cohort now exceeds 200 cases.
- ADNI NPC data in National Alzheimer's Coordinating Center format are available through the Laboratory of Neuro Imaging.
- Digitized slide files from the ADNI NPC will be available in 2025.
- Requests for ADNI brain tissue samples can be submitted online for ADNI/National Institute on Aging evaluation.
- Clinical diagnoses of Alzheimer's disease (AD)/AD and related dementias (ADRD) do not always predict *post mortem* neuropathology.
- Neuropathology is essential for the development of novel AD/ADRD biomarkers.

## 1 BACKGROUND

Alzheimer's disease (AD) neuropathologic change (ADNC), defined by parenchymal deposits of amyloid beta (Aβ) peptide (“plaques”) and intraneuronal aggregates of hyperphosphorylated tau protein (“neurofibrillary tangles”), is the leading cause of dementia late in life but is not the only cause. The neuropathological processes responsible for cognitive decline later in life are diverse, have overlapping phenotypes, and often coexist. Even dominantly inherited AD (caused by pathogenic variants in *APP*, *PSEN1*, and *PSEN2* genes), which is generally considered a “pure” form of AD, shows heterogeneity between cases and often includes Lewy body pathology (intraneuronal aggregates of alpha‐synuclein) and TAR DNA binding protein 43 (TDP‐43) pathology (intracellular aggregates of TDP‐43).^1^, ^2^ If we are to prevent and treat AD and related dementias (ADRD), we must (1) understand the patterns and combinations in which neurodegenerative pathologies can appear in individuals, (2) improve our ability to detect these specific neuropathologic changes during life, and (3) develop effective means to evaluate candidate therapeutics in clinical trials.

These goals require new biomarker discovery, validation, and implementation. What may be less intuitive to some is that neuropathologic examinations are absolutely essential to achieve them. Although effective biofluid and neuroimaging biomarkers for ADNC (plasma and cerebrospinal fluid [CSF] variants of Aβ and tau; positron emission tomography (PET) imaging of tracers that bind Aβ and tau aggregates) are now in use in clinical trials (eg, refs. 3, 4, 5, 6, 7, 8, 9), only neuropathologic examination can identify and stage other common neuropathologies – such as Lewy body disease and TDP‐43 proteinopathy – that can impair cognition, impact other biomarker measurements, such as those for neurodegeneration and neuroinflammation (eg, neurofilament light chain [NfL], CSF YKL‐40, glial fibrillary acidic protein, sTREM2, magnetic resonance imaging [MRI]),^10^, ^11^, ^12^, ^13^, ^14^, ^15^, ^16^ and influence clinical trial outcomes. *Post mortem* examination also enables the creation of well‐characterized brain tissue resources that can support new biomarker discovery/validation efforts, deepen our understanding of the pathophysiology associated with all forms of neurodegeneration, and study, in‐depth, the effects of disease‐modifying treatments on the brain.

This is the perspective of the Alzheimer's Disease Neuroimaging Initiative (ADNI) Neuropathology Core (NPC): our mission is to share “gold standard” neuropathologic data and high‐quality brain tissue specimens from autopsy‐consented ADNI participants with the scientific community to advance the field of AD/ADRD biomarker research and development.

In this report, the ADNI NPC describes its relevant history, organization, methods, and offerings; the neuropathological features of its first 100 completed brain donation assessments; how those features correlate with clinical diagnoses; the implications of these correlative findings for ADRD clinical trials and diagnostic biomarker use in the clinic; and its anticipated contributions to ADRD research in ADNI4 and beyond.

## 2 METHODS

### 2.1 ADNI brain donation program

When ADNI was launched in 2004, it was designed as a short‐term, 5‐year study that did not include neuropathologic assessment and the establishment of a brain tissue resource among its goals. Hence, ADNI began without a NPC. Nevertheless, with an original enrollment of *n* = 188 with AD dementia (ADD), *n* = 402 with mild cognitive impairment (MCI), and *n* = 229 “elderly controls,” some participant deaths were recorded even during the first 3 years of the study. Recognizing the importance of neuropathologic confirmation for AD biomarker‐related studies, John C. Morris, the ADNI site leader at Washington University, submitted a competing revision to the National Institute on Aging (NIA) on behalf of the parent ADNI grant that proposed establishing a NPC for ADNI. The revision was awarded in 2007, and the ADNI NPC began with Morris as Core Leader and Nigel J. Cairns, PhD, as the neuropathologist; the performance site was Washington University. However, this early NPC was not included in ADNI's main protocol document, and brain donation was not included in participant enrollment consent forms. Additionally, it lacked a full‐time NPC coordinator, the authority and power to track participants after withdrawal from the study for eventual brain donation, and sufficient budget to support the coordinator's effort and reimburse autopsy costs at participating sites. These conditions continued even as ADNI's cohort matured and grew, with the addition of *n* = 131 “early MCI” participants during ADNI‐GO (2009 to 2011) and *n* = 790 additional participants (*n* = 151 dementia, *n* = 345 MCI, *n* = 294 “elder controls”) during ADNI2 (2011 to 2016). Brain donation was not included in the ADNI participant enrollment consent until just before the inception of ADNI3 (2016 to 2021, during which *n* = 72 ADD, *n* = 237 MCI, and *n* = 374 “elder controls” were enrolled) and participant tracking until expiration – often disallowed by local site Institutional Review Boards (IRBs) – was not possible until ADNI's contract research organization (the Alzheimer's Therapeutic Research Institute [ATRI]) supported it in 2017. Unfortunately, this tracking was initially restricted to participants with ADD and was not supported by funding from ADNI. By 2019, only 20 of the 59 ADNI sites with autopsy capability had secured IRB approval to track participants after withdrawal from active participation. Most of these 20 sites already had active brain donation programs of their own, supported by local Alzheimer's Disease Research Centers (ADRCs). Indeed, since the beginning of ADNI, many ADNI participants have been co‐enrolled as ADRC participants. Although co‐enrollment has undoubtedly increased the number of successful brain donations by ADNI participants to sites, their co‐enrollment in ADNI often went unrecognized at the time of expiration, so specimens were not always shared with the ADNI NPC. As a consequence of all these historical challenges, the number of brain donations secured by the ADNI NPC by the end of 2018 was limited to 94, contributed by just 30 ADNI sites. In August 2018, Richard J. Perrin, MD, PhD, succeeded Nigel Cairns as the neuropathologist and co‐leader of the ADNI NPC.

**RESEARCH IN CONTEXT**

1. **Systematic review**: The authors reviewed the literature relevant to Alzheimer's Disease Neuroimaging Initiative (ADNI), the ADNI Neuropathology Core, neuropathologic diagnostic criteria, and clinicopathologic studies of ADRD using traditional (eg, PubMed) sources and meeting abstracts and presentations.
2. **Interpretation**: Our findings support the hypothesis that clinical diagnoses of Alzheimer's disease and related dementias (ADRD) in ADNI do not always accurately predict *post mortem* neuropathology. These findings are consistent with other population studies of ADRD in older adults. Our findings further suggest that biomarkers are needed to improve *ante mortem* diagnosis and that neuropathologic examinations are important for biomarker discovery and development.
3. **Future directions**: Future clinicopathological correlations of the ADNI participant brain donor cohort as it grows will expand, confirm, or refute these findings.

Fortunately, in 2019, with support from ADNI and NIA leadership, circumstances began to improve. The NPC conducted a formal ADNI‐wide survey to identify common and uncommon obstacles to successful brain donation programs at ADNI sites and secured two NIA‐sponsored administrative supplements to address them. These supplements enabled the hiring of ADNI's first full‐time NPC coordinator, increased the cap for reimbursement of site expenses related to autopsy and tissue provision to ADNI, and increased ATRI's oversight and monitoring of site participation in the brain donation program, emphasizing to sites that brain donation was an expected standard for the completion of study metrics. It also supported a mini‐grant program administered by the NPC through which ADNI sites could apply for supplemental funding earmarked to address self‐identified site‐specific obstacles to successful brain donation, including support for coordinator effort at individual ADNI sites. Twenty‐six sites applied for and received funding through this initiative. ADNI sites without active brain donation programs received NPC assistance to develop a plan. ADNI sites without IRB approval to seek consent for brain donation were assisted and aggressively encouraged to obtain approval. ADNI sites with active autopsy programs were encouraged to support the efforts of the ADNI NPC and to share tissue specimens from dually enrolled participants. Many ADNI2 participants who had been lost to follow‐up were located and re‐enrolled in a new ADNI3 “brain donation only” program, spearheaded by ADNI's new NPC coordinator in conjunction with ATRI. As a result of all these efforts, the ADNI Brain Donation Network now includes 56 participating sites, and, by the end of ADNI3, over 570 living ADNI participants had consented to brain donation. Another breakthrough came in 2021 when the National Centralized Repository for Alzheimer's Disease and Related Dementias (NCRAD), by matching DNA profiles, identified *n* = 49 ADNI participants who, unexpectedly, had neuropathology data in the National Alzheimer's Coordinating Center (NACC) database, indicating that they were ADRC/ADNI co‐enrollees whose brain donations to ADRCs had not yet been shared with the ADNI NPC.^17^ When asked, all the involved ADRCs (*n* = 18) graciously agreed to share samples from these cases with the ADNI NPC. Reflecting all these advancements, the ADNI NPC Brain Donation Cohort has expanded very quickly; it now includes 224 cases, and this number continues to grow.

To oversee this resource, the ADNI and the NIA established a Neuropathology Resource Allocation Review Committee (NP‐RARC), which serves to review applications for the use of ADNI brain tissue specimens and to make recommendations to the NIA about sample allocation.

In 2024, with ADNI4 secured and under way, the ADNI NPC transitioned its leadership from Morris to Perrin.

### 2.2 Brain donation – participant registration, consent, and monitoring

Because Advarra, which has served as the central IRB for ADNI since the start of ADNI4, considers brain donation to fall outside the regulatory definition of “human subjects research,” ADNI4 participants no longer provide legal consent for autopsy through ADNI; instead, they may register with ADNI as willing brain donors, with legal consent to be obtained from next‐of‐kin or power of attorney according to local laws and regulations upon the participant's death. Newly enrolled ADNI4 participants are asked to consider registering for brain donation upon enrollment. Those new participants – and ADNI3 participants actively continuing in ADNI4 – who remain undecided are asked again at subsequent annual visits until they decline or assent. Some ADNI3 participants who discontinued their active participation in ADNI have, instead, enrolled in ADNI4's “brain donation only” program. Brain donor registrants are monitored with bi‐annual telephone contacts to confirm geographic location and participant/caregiver awareness. Sites are encouraged to plan logistics for each participant's brain donation well in advance of anticipated expiration and to update that plan after each telephone check, as needed. Upon the death of a participant who has registered for brain donation (or remains undecided), family/caregiver notification of the participant's ADNI site coordinator is intended to initiate the donation protocol, which includes seeking/obtaining legal consent and carrying out brain removal as soon as is practical, to minimize *post mortem* interval (PMI; between death and brain tissue fixation/freezing) and any delay of funeral arrangements. No upper limit is set on PMI, as even extended PMIs still permit histologic analysis, but PMI < 12 to 24 h is preferred to maximize tissue quality for biochemical and molecular studies.

### 2.3 Brain tissue – macroscopic assessment, processing, histological sampling

When possible, the ADNI NPC prefers receiving brain donations in complete form. For most cases, this includes a sagittally bisected specimen with an intact, formalin‐fixed left hemibrain and a frozen right hemibrain – with cerebellum, brainstem, and supratentorial components frozen separately but intact at −80°C, or frozen after they are sliced (supratentorial structures, coronally at 1‐cm intervals; cerebellum, sagittally at 1‐cm intervals; brainstem, axially, at ∼0.5‐cm intervals) and, ideally, photographed. The appropriate approach to freezing depends on the expertise and capability of the tissue procurement team involved in the case. This approach permits the ADNI NPC to assess macroscopic features and to ensure completely uniform neuroanatomic sampling across cases – for both neuropathologic assessment (histology) and frozen sample preparation for research studies.

However, the ADNI NPC acknowledges that many ADNI participants registered for brain donation are co‐enrolled in other studies that have independent brain donation programs and neuropathology protocols. For such cases, when possible, the ADNI NPC encourages the involved center to communicate with the ADNI NPC in advance to ensure that the protocols for both studies can be accommodated as closely and completely as possible, with minor modifications if absolutely necessary. When a brain is processed and evaluated first at an outside center, the ADNI NPC requests a standard set of ∼17 fixed tissue blocks (either “wet,” processed into paraffin, or represented by slide‐mounted 6‐µm‐thick unstained paraffin sections when a sampled area has insufficient tissue to support block sharing) and a standard limited set of ∼five 1‐cm‐thick frozen tissue slabs (1: frontal lobe to include striatum [coronal]; 2: frontal and temporal lobe at level of mammillary body [coronal]; 3: temporal and parietal lobes at level of lateral geniculate nucleus [coronal]; 4: occipital lobe to include the calcarine sulcus [coronal]; 5: cerebellar hemisphere to include dentate nucleus [parasagittal/radial/coronal]). Additional histologic blocks or unstained slides representing focal abnormalities unique to a case (eg, infarcts, hemorrhages, neoplasms) that appear outside these prescribed sampled areas are also requested. Macroscopic descriptions written by qualified personnel and/or photographs of intact and sliced specimens, when available, are also appreciated. When donations from co‐enrolled participants are, instead, initially processed by the ADNI NPC, we strive to provide similar representative samples and macroscopic descriptions/photographs to the outside center, accommodating all provided outside protocol instructions, when possible.

### 2.4 Histology/immunohistochemistry (IHC)

**FIGURE 1:**
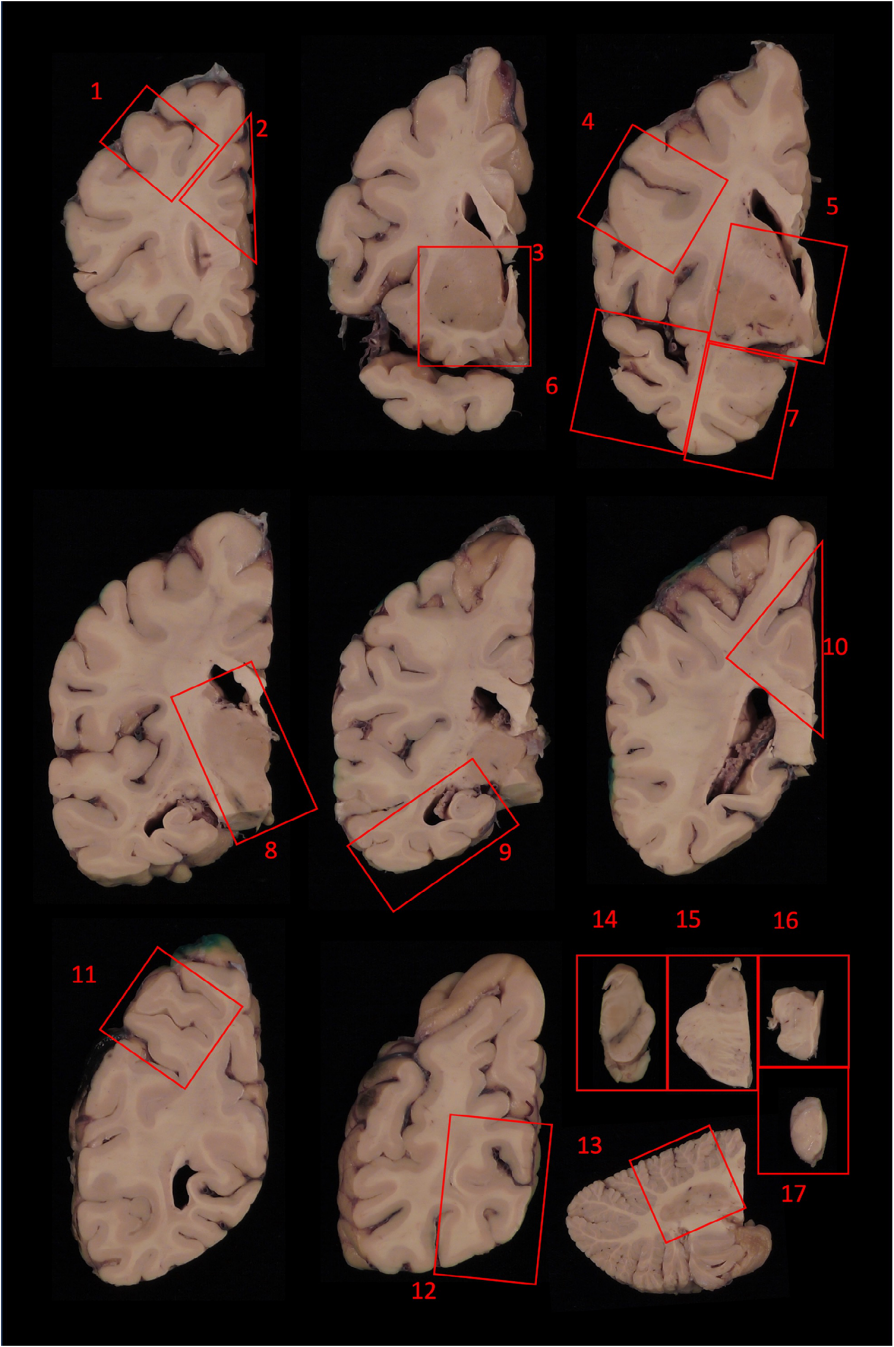
Schematic of standardized ADNI NPC histologic sampling. After fresh sagittal bisection, freezing of the right hemibrain, and thorough formalin fixation of the left hemibrain, the left hemibrain is sliced (supratentorial structures, coronally; cerebellum, sagittally; brainstem and associated cervical spinal cord, axially), and at least 16 regions are sampled, with cervical spinal cord included as a 17th region, when available. Other areas are sampled as needed to enable histological examination of any macroscopic abnormalities outside the standard areas. Each histologic sample is processed into paraffin wax and 6‐µm glass‐mounted sections of each are subsequently stained with H&E and by IHC for beta‐amyloid, phosphorylated tau, alpha‐synuclein, and pTDP‐43 as described in Methods (Section 2.4). Areas sampled: 1: middle frontal gyrus; 2: anterior cingulate gyrus; 3: striatum with nucleus accumbens and olfactory cortex; 4: precentral gyrus; 5: pallidum and basal forebrain with nucleus basalis of Meynert; 6: superior and middle temporal gyri; 7: amygdala with ambient, fusiform and inferior temporal gyri; 8: thalamus with subthalamic nucleus; 9: hippocampus with parahippocampal gyrus, fusiform gyrus, inferior temporal gyrus; 10: posterior cingulate gyrus and precuneus; 11: angular gyrus (parietal lobe); 12: calcarine sulcus and parastriate cortex; 13: dorsal cerebellar cortex with dentate nucleus; 14: midbrain with red nucleus; 15: pons with locus coeruleus; 16: medulla with dorsal motor nuclei and inferior olive; 17: cervical spinal cord (when available). ADNI, Alzheimer's Disease Neuroimaging Initiative; H&E, hematoxylin and eosin; IHC, immunohistochemistry; NPC, Neuropathology Core.

**FIGURE 2:**
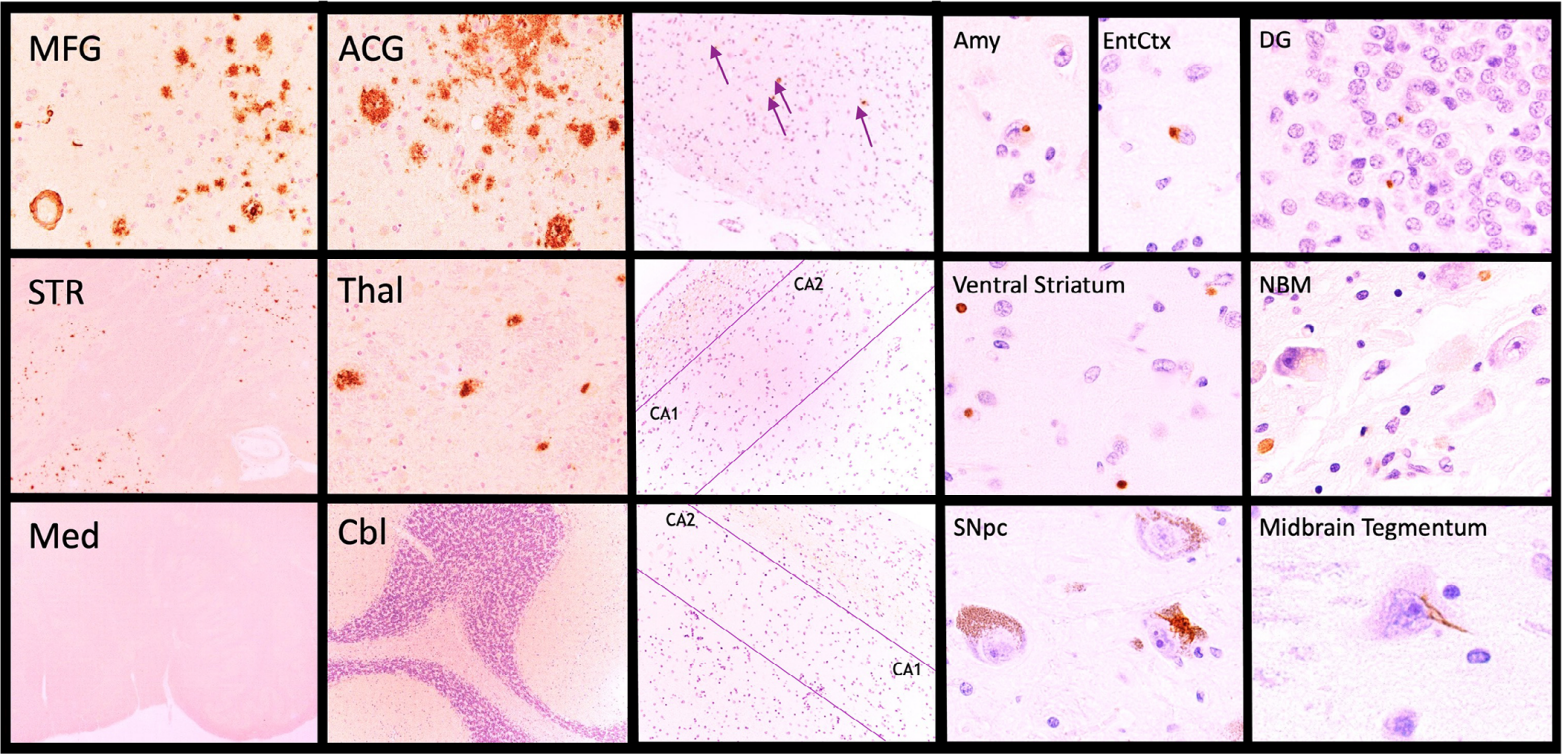
ADNI participant diagnosed clinically with moderate Alzheimer's disease dementia and mild cerebrovascular disease. Columns 1 and 2 illustrate the distribution of amyloid beta plaques restricted to neocortex, striatum, and thalamus and absent from brainstem and cerebellum, consistent with Thal phase 3. Column 3 illustrates meager tau‐immunoreactive NFTs (arrows) largely restricted to the entorhinal cortex (upper panel), consistent with Braak NFT stage I, and severe neuronal loss in the pyramidal layer (bounded by lines) without NFTs in the left (center panel) and right (lower panel) hippocampal formations, consistent with bilateral HS. Columns 4 and 5, complementing the finding of bilateral HS, illustrate a broad distribution of pTDP‐43 immunoreactive neuronal cytoplasmic inclusions that nevertheless spares the frontal cortex, consistent with Josephs stage 5 and LATE‐NC stage 2. ACG, anterior cingulate gyrus; ADNI, Alzheimer's Disease Neuroimaging Initiative; Amy, amygdala; CA1 and CA2, hippocampal pyramidal cell subfields; Cbl, cerebellum; DG, dentate gyrus; EntCtx, entorhinal cortex; HS, hippocampal sclerosis; LATE‐NC, limbic‐predominant age‐related TDP‐43 proteinopathy ‐ neuropathologic change; Med, medulla oblongata; MFG, middle frontal gyrus; NBM, nucleus basalis of Meynert; NFT, neurofibrillary tangles; SNpc, substantia nigra pars compacta; STR, striatum; Thal, thalamus.

**FIGURE 3:**
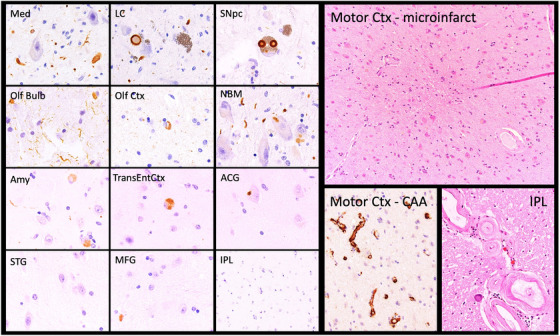
ADNI participant diagnosed clinically with moderate ADD and mild cerebrovascular disease (continued from Figure 2). The 12‐panel array on the left illustrates the distribution of Lewy bodies and Lewy neurites immunoreactive for phosphorylated alpha‐synuclein, most consistent with the limbic (transitional) stage^22^,^23^ and Braak stage 3 or 4, likely to contribute to impaired cognition, but generally inconsistent with symptomatic idiopathic Parkinson's disease. The upper right panel illustrates an H&E‐stained microinfarct identified by chance within the precentral gyrus; as a presumptive representative of other similar undetected neocortical microinfarcts, even an isolated lesion such as this has been shown to correlate with additive cognitive impairment.^19^ The lower right panels illustrate the severe, widespread CAA in this case, even affecting capillaries within the precentral gyrus, and its regionally severe arteriolosclerosis; either of these vasculopathic processes, or both, may have increased the likelihood of cortical microinfarction. ACG, anterior cingulate gyrus; ADD, AD dementia; ADNI, Alzheimer's Disease Neuroimaging Initiative; Amy, amygdala; CAA, cerebral amyloid(‐beta) angiopathy; H&E, hematoxylin and eosin; Med, medulla oblongata; IPL, inferior parietal lobule; LC, locus coeruleus; MFG, middle frontal gyrus; Motor Ctx, primary motor cortex (precentral gyrus); NBM, nucleus basalis of Meynert; Olf bulb, olfactory bulb; Olf ctx, olfactory cortex; TransEntCtx, transentorhinal cortex; SNpc, substantia nigra pars compacta; STG, superior temporal gyrus.

From each of 17 formalin‐fixed, paraffin‐embedded (FFPE) tissue samples per case (Figure 1), the ADNI NPC stains glass slide‐mounted 6‐µm‐thick sections with hematoxylin and eosin (H&E) and by standard (3,3′‐diaminobenzidine [DAB]) IHC using a Leica Bond III autostainer and antibodies for Aβ (10D5, Eli Lilly, Indianapolis, IN, USA); phosphorylated tau (mouse monoclonal PHF‐1, Feinstein Institute for Medical Research, Manhasset, NY, USA); phosphorylated alpha‐synuclein (rabbit monoclonal, Catalog No. CG1656, Cell Applications, San Diego, CA, USA) or non‐phosphorylated alpha‐synuclein (mouse monoclonal LB509, MilliporeSigma Burlington, MA, USA); and phosphorylated TAR DNA binding protein 43 (pTDP‐43) (mouse monoclonal pTDP‐43 [pS409/410], Cosmo Bio USA, Carlsbad, CA, USA). Photomicrographs representing these staining preparations are included in Figures 2 and 3.

### 2.5 Histologic semi‐quantitative assessments and diagnostic/staging criteria

Every slide is reviewed systematically by an expert neurodegenerative neuropathologist to generate semi‐quantitative scores for observed neurodegenerative features including neuronal loss and gliosis; features that characterize ADNC (Aβ plaques, neuritic plaques, and neurofibrillary tangles); lesions that characterize other common and uncommon neurodegenerative proteinopathies (eg, other tau‐immunoreactive neurons, neuronal inclusions, glial inclusions, and cell processes; alpha‐synuclein immunoreactive Lewy bodies and glial cytoplasmic inclusions; pTDP‐43‐immunoreactive inclusions and cellular processes); cerebral vasculopathies (eg, arteriolosclerosis, cerebral amyloid angiopathy, and mineralization); and other relevant findings (eg, white matter pallor, microinfarctions, microhemorrhages, and masses). These regional semi‐quantitative scores are then evaluated according to established historical and contemporary neuropathologic criteria to render all appropriate neuropathologic diagnoses and stages for each case. The neuropathologic findings from each case are included in a comprehensive *post mortem* neuropathologic research report prepared by the ADNI NPC and provided to relevant ADNI site principal investigators (PIs); these can be shared with the participant's next‐of‐kin or legal power of attorney as appropriate. The neuropathologic data obtained from each case are also used to complete updated standardized NACC neuropathology forms (according to appropriate coding guidebook instructions); this approach permits direct harmonization of data from the ADNI neuropathology cohort (available through the Laboratory of Neuro Imaging [LONI]) with those from all ADRC cohorts (available through the NACC database).

### 2.6 Digital ADNI NPC histopathology resource

In addition to the NACC‐compatible neuropathology data (described above) currently available through LONI, the ADNI NPC will soon be providing free access to its entire digitized histologic slide collection. Using a Leica Aperio AT2 Digital Scanner, purchased jointly by ADNI and the Washington University Knight ADRC, the ADNI NPC has begun to generate and annotate an unabridged library of digitized ADNI histology slides; this resource is expected to become available through the LONI portal by 2025.

### 2.7 Brain tissue sample sharing

The ADNI NPC biospecimen resource includes glass‐mounted unstained FFPE sections, frozen tissue samples, and “wet” formalin‐fixed tissue samples from neuropathologically assessed ADNI participants. Samples from this resource are accessible by non‐ADNI investigators through a formal application process described on the ADNI website (https://adni.loni.usc.edu). Successful requests will leverage the unique and extensive non‐neuropathological ADNI dataset associated with these samples to address questions relevant to the ADNI mission and will demonstrate technical feasibility with preliminary data. Serious preliminary inquiries are initially reviewed by the ADNI NP‐RARC and NIA for feasibility before a full‐length application is invited. Invited applications are evaluated by the ADNI NP‐RARC, which makes a recommendation to the NIA. Ultimate approval is provided by the NIA. Samples provided in support of approved requests may be used only by the original intended recipients and only for the scientific inquiry proposed and approved. Data generated from their use must be made publicly available after publication according to NIH guidelines, and ADNI's contribution must be acknowledged in any publications or presentations of findings.

## 3 RESULTS

To begin to understand the potential for neuropathology data to influence ADNI research, we undertook a clinicopathological assessment of the first 100 completed ADNI cases.

### 3.1 Clinical characteristics

The first 100 cases, described in Table 1, represent a mean age of 82.7 years (range: 59 to 97), are 76% male, and include a range of APOE genotypes (even with limited genotyping data available). As expected for a cohort initially assembled to favor the representation of symptomatic ADD, most cases (*n* = 80) carried that clinical diagnosis at expiration, with *n* = 8 also carrying a second clinical diagnosis (DLB, Parkinson's disease [PD], parkinsonism, vascular dementia [stroke/cerebrovascular disease]); among those without ADD, three participants were considered to have MCI due to AD, five were considered to be cognitively unimpaired, one had progressive supranuclear palsy, one had normal pressure hydrocephalus and parkinsonism, one had vascular dementia and parkinsonism, and one had DLB. Corresponding with this distribution, Clinical Dementia Rating^®^ (CDR) scores at expiration spanned the entire range but were predominantly >0 (*n* = 88, with no CDR score available for *n* = 7); most participants exhibited moderate or severe dementia and received CDR scores of 2 or 3.

**TABLE 1:** Clinical characteristics of first 100 autopsy cases assessed by ADNI.

*CDR at expiration*	*N*
CDR 0	5
CDR 0.5	10
CDR 1	8
CDR 2 or 3	70
Unknown	7

*APOE genotype* ^a^	*N* (% among available^a^)
ε4/ε4	8 (18)
ε3/ε4	20 (44)
ε3/ε3	36 (16)
ε2/ε4	2 (1)
Unknown	55

Clinical diagnosis at expiration	*N*
Cognitively unimpaired	5
MCI due to AD	3
ADD only	80
ADD + other	8
ADD + DLB	1
ADD + PD	2
ADD + Parkinsonism	3
ADD + Parkinsonism + stroke	1
ADD + cerebrovascular disease	1
PSP	1
Normal pressure hydrocephalus + Parkinsonism	1
Vascular dementia + Parkinsonism	1
DLB	1

*Note*: age: mean 82.7 years (range 59 to 97 years); sex: 76% male.

Abbreviations: ADD, Alzheimer's disease dementia; AD, Alzheimer's disease; ADNI, Alzheimer's Disease Neuroimaging Initiative; APOE, apolipoprotein E gene; CDR, Clinical Dementia Rating; DLB, dementia with Lewy bodies; MCI, mild cognitive impairment; PD, Parkinson's disease; PSP, progressive supranuclear palsy.

^a^ *APOE* data are available for only 45% of cases.

### 3.2 Frequencies of neuropathologic changes

Neuropathologic examination of the cohort revealed a broad spectrum of neuropathologies with variation in severity (Table 2). Not unexpectedly, ADNC (staged according to NIA‐Alzheimer's Association [AA] criteria)^18^ was quite common. Correlating ADNC with ADD (Table 3) revealed that only four of 76 individuals with high or intermediate ADNC – considered to be sufficient to account for clinical dementia – had not been diagnosed with ADD even at the end of life; this suggests a 95% sensitivity for the clinical prediction of symptomatic ADNC. Among all 91 individuals diagnosed with ADD (with or without an additional clinical diagnosis), 72 had high or intermediate ADNC, indicating a 79% specificity for the clinical prediction of symptomatic ADNC. The other *n* = 19 diagnosed with ADD had “low” ADNC or no (“not”) ADNC, indicating a 21% false positive rate. Finally, four of nine individuals who were not diagnosed with ADD at the end of life were found to have high or intermediate ADNC at autopsy, suggesting an ∼44% false negative rate for the clinical prediction of symptomatic ADNC at or near expiration in the ADNI cohort. These results suggest that clinical presentation alone (as assessed by ADNI site clinicians within a research cohort preselected to enrich for ADD) is a reasonable but imperfect predictor of ADNC at autopsy.

**TABLE 2:** Frequencies of neuropathologic changes.

Neuropathologic change (staging)	Distribution of cases by stage (*n*)					
	Severe			Mild	Absent	Missing (*n*)
ADNC (high, intermediate, low, not)	68	8		19	5	0
PART (definite: Braak IV, III, II, I, not)	1	0	3	1	95	0
LBD (diffuse, limbic, BS, amy/olf, none)	21	11	4	15	49	0
AGD (stage III, II, I, 0)	6	5		13	76	0
TDP‐43/LATE‐NC (Josephs 5–6, 3–4, 1–2, 0)	5	17		22	53	3^c^
HS (yes/no)	8				92	0
Infarction(s) (macro^a^/micro/none)	7		24^b^		72^a^	1^a^
Atherosclerosis (severe, mod, mild, none)	4	16		46	14	20
Arteriolosclerosis (severe, mod, mild, none)	7	24		64	5	0
CAA (severe, moderate, mild, none)	18	22		43	17	0
Aging‐related tau astrogliopathy (yes/no)	55				41	4
Progressive supranuclear palsy (yes/no)	2				98	0
FTLD‐TDP (yes/no)	1				99	0

Abbreviations: ADNC, Alzheimer's disease neuropathologic change; AGD, argyrophilic grain disease; amy/olf, amygdala‐predominant or olfactory bulb‐limited; CAA, cerebral amyloid angiopathy; FTLD‐TDP, frontotemporal lobar degeneration with TDP‐43‐immunoreactive pathology; LATE‐NC, limbic‐predominant age‐related TDP‐43 encephalopathy–neuropathologic change; LBD, Lewy body disease; Mod, moderate; PART, primary age‐related tauopathy; TDP‐43, TAR DNA‐binding protein 43.

^a^ One case has no macroscopic description available but does have histology–with microinfarctions.

^b^ Some participants had both macro‐ and microinfarctions–72 participants had neither.

^c^ Three very old cases (2008/2009) were never stained for TDP‐43.

**TABLE 3:** Frequency of ADNC stages predicted by clinical diagnosis at expiration.

	AD neuropathologic change		
Clinical diagnosis at expiration	High/intermediate (*n* = 76)	Low (*n* = 19)	Not (*n* = 5)
**ADD only** ^a^ **(*n* = 83)**	68	13	2
**ADD + other** ^b^ **(*n* = 8)**	4	3	1
**Not ADD** ^c^ **(*n* = 4)**	3	1	0
**Normal (*n* = 5)**	1	2	2

Abbreviations: ADD, Alzheimer's disease dementia; ADNC, Alzheimer's disease neuropathologic change.

^a^ “ADD only” includes ADD and “MCI due to AD.”

^b^ “ADD + other” includes: parkinsonism = 3; Parkinson's disease = 2; dementia with Lewy bodies = 1; vascular = 1; stroke + parkinsonism = 1.

^c^ “Not ADD” includes: DLB = 1; PSP = 1; vascular dementia + parkinsonism = 1; NPH + parkinsonism = 1.

One potential cause of the imperfect clinicopathologic correlation between ADD and ADNC is the existence of “co‐pathologies.” Indeed, in this cohort, 65 (85%) of the 76 participants with high or intermediate ADNC had one or more additional pathologies, including (in order of frequency): “aging‐related tau astrogliopathy” (ARTAG), LBD, “limbic‐predominant age‐related TDP‐43 proteinopathy–neuropathologic change” (LATE‐NC), vasculopathy, argyrophilic grain disease (AGD), and/or hippocampal sclerosis (HS) (Table 4). Admittedly, in some of these cases, the co‐pathologies observed are likely insufficient to have impaired function. However, with careful consideration of the staging/severity of their co‐pathologies in the context of published reports predicting their likely independent contributions to cognitive impairment, it would appear that approximately 60% (46/75) of cases with high/intermediate ADNC and dementia (CDR > 0) did harbor moderate (*n* = 16) or severe (*n* = 30) co‐pathologies (eg, infarcts,^19^ AGD stages II or III,^20^, ^21^ diffuse neocortical or limbic [diffuse/limbic] LBD,^22^, ^23^ LATE‐NC, and HS)^24^ with strong potential to contribute to dementia (Table 5).

**TABLE 4:** Frequencies of neuropathologic findings in entire cohort and in association with ADNC.

Neuropathologic finding(s)	Percentage of cases (*n*)	Percentage of high/intermediate ADNC
ADNC only (high/intermediate)	11 (11/100)	15 (11/76)
ADNC (high/intermediate) + “others”	65 (65/100)	85 (65/76)
+ ARTAG	62 (40/65)	53 (40/76)
+ Synucleinopathy (LBD)	58 (38/65)	50 (38/76)
+ TDP‐43/LATE	52 (34/65)	45 (34/76)
+ Vascular	32 (21/65)	28 (21/76)
+ AGD	18 (12/65)	16 (12/76)
+ HS	6.2 (4/65)	5 (4/76)
Low ADNC + broad range of “others”^a^	19 (19/100)	N/A
Not ADNC (AGD/PART/Infarcts/LATE‐NC/LBD)^b^	5 (5/100)	N/A

Abbreviations: ADNC, Alzheimer's disease neuropathologic change; AGD III, argyrophilic grain disease, Saito stage III; ARTAG, aging‐related tau astrogliopathy; LBD, Lewy body disease; PART, primary age‐related tauopathy; TDP‐43, TAR DNA‐binding protein 43.

^a^ Low ADNC “others” includes: FTLD‐TDP (*n* = 1); PSP (*N* = 2); LBD (*N* = 12); HS (*N* = 4); LATE‐NC (*N* = 9); AGD (*N* = 9); infarcts (*N* = 5); ARTAG (*N* = 12).

^b^ One CDR 3 had AGD III; one CDR 0.5 had AGD III and PART and ARTAG; one CDR 0.5 had microinfarcts and LATE‐NC and brainstem‐predominant LBD; two were CDR 0.

**TABLE 5:** Likelihood of contribution of non‐ADNC findings to dementia in 75 cases with both dementia (CDR > 0) and high/intermediate ADNC.

Likelihood^a^ of non‐ADNC contribution to dementia	Additional ”significant’” non‐ADNC findings	Percentage of cases (*n*)
None	None (high/intermediate ADNC only)	14.7 (11/75)
Minimal	ARTAG or brainstem‐predominant LBD only	10.7 (8/75)
Low	ALB or AGD I or TDP‐Josephs stage 1 and 2	13.3 (10/75)
Moderate	Infarct/s or TDP‐Josephs stage 3 and 4 or AGD II	21.3 (16/75)
High	Diffuse/limbic LBD or HS or AGD II/III	40.0 (30/75)

Abbreviations: ADNC, Alzheimer's disease neuropathologic change; AGD, argyrophilic grain disease; ALB, amygdala‐predominant LBD; ARTAG, aging‐related tau astrogliopathy; CDR, Clinical Dementia Rating; HS, hippocampal sclerosis.; LBD, Lewy body disease; TDP, TAR DNA‐binding protein 43.

^a^ ADNI NPC opinion, guided by refs. 12, 19, 20, 21, 22, 23, 24.

Another potential cause of this imperfect clinicopathologic correlation is deviation from the clinical presentation that might be retrospectively “predicted” from the neuropathology. For example, one participant with intermediate ADNC (NIA‐AA ADNC score: A2, B2, C0 [Braak neurofibrillary tangle [NFT] stage IV], with only AGD stage I, mild ARTAG, moderate CAA, and moderate arteriolosclerosis as co‐pathologies) was determined to be CDR0 at expiration. Deviation from anticipated clinical presentation can also include “clinical mimicry.” Examination of 15 cases who received a “pure” clinical diagnosis of ADD but exhibited “low” or “not” ADNC at autopsy (Table 3) revealed that each had one or more non‐ADNC neuropathologies considered sufficient to impair cognition: limbic or diffuse neocortical LBD, HS, LATE‐NC, frontotemporal lobar degeneration with TDP‐43‐immunoreactive pathology, AGD stage II or III, microinfarcts, and/or moderate arteriolosclerosis and substantial white matter pallor (supporting a diagnosis of “subcortical arteriolosclerotic leukoencephalopathy”). One of these cases with ADD, low ADNC, and multiple non‐AD neurodegenerative pathologies is represented in detail in Figures 2 and 3. Four other cases represented in Table 3 with clinical diagnoses of ADD plus an additional neurological disorder (eg, PD, DLB, cerebral vascular disease) – but who were likewise found to have “low” or “not” ADNC – also exhibited one or more of the following: limbic or diffuse neocortical LBD, LATE‐NC, HS, PART (Braak NFT stage IV), AGD stage II or III, microinfarcts, and macroinfarcts. Conversely, four of the nine cases with non‐ADD clinical diagnoses were determined to have high/intermediate ADNC. Of these four, one (diagnosed with normal pressure hydrocephalus and parkinsonism) had intermediate ADNC, progressive supranuclear palsy neuropathologic change (Kovacs stage 3),^25^ LATE‐NC stage 2 (Josephs stage 3),^26^, ^27^ mild to moderate vasculopathy, and a remote/subacute medullary infarct; another (diagnosed with DLB) had only high ADNC and severe CAA; another (diagnosed with vascular dementia and parkinsonism) had high ADNC, mild to moderate vasculopathy, and microinfarcts; the fourth case (described as cognitively normal) had intermediate ADNC (NIA‐AA score: A2, B2, C0 [Braak NFT stage IV]), AGD stage I, and moderate arteriolosclerosis and CAA. Why these pathologies manifested unpredictably in these cases is unclear. Nevertheless, they illustrate the critical importance and potential benefit of using biomarkers – not just to diagnose and stage ADNC but also to detect and stage other non‐ADNC pathologies.

## 4 DISCUSSION

This study of the first 100 ADNI participant neuropathologic examinations is somewhat small and preliminary. Nevertheless, its findings resemble those from other similar cohorts^28^, ^29^, ^30^, ^31^, ^32^ – perhaps with subtle differences, given the emphasis of the original ADNI recruitment effort to enroll individuals with moderate to severe ADD. These studies all demonstrate that clinical diagnoses do not always accurately predict *post mortem* neuropathologic findings. Our study, which used retrospective end‐of‐life clinical diagnoses and not simply the diagnosis at the last clinical assessment, emphasizes that these clinicopathological mismatches cannot be attributed to a long interval between clinical diagnosis and autopsy. These imperfect predictions are not the fault of the clinicians; they reflect the heterogeneity and diversity of neuropathologic changes in the population and the variability with which those changes can influence neurological function. Regardless, they emphasize the importance of neuropathologic examinations to provide “gold standard” diagnoses of neurological diseases. They also demonstrate that sensitive and specific biomarkers will be essential for improving the accurate identification of all neuropathological changes relevant to dementia during life. Accurate and comprehensive *ante mortem* diagnosis is key for designing effective and efficient clinical trials for ADRD; it will also be critical for administering the most appropriate therapeutic(s) to patients, as more disease‐modifying therapies receive US Food and Drug Administration approval.

Supporting ADRD biomarker research is, of course, ADNI's *raison d'être*. Since its launch, ADNI has made essential contributions to the discovery and development of many of the leading biofluid and neuroimaging biomarkers for AD – even without the benefit of a robust neuropathology database. Moving forward, however, as the world stands witness to the first successful disease‐modifying clinical trials for AD,^3^, ^33^ as non‐AD neuropathologies are targeted by molecular studies, candidate biomarkers, and novel therapeutics, and as the study of demographic and cultural diversity in ADRD research receives its due, it is clear that high‐quality neuropathology data and brain tissue specimens will be critical for rapid progress and ADNI's success.

Harbingers of that future are in evidence. Already, neuropathology data from ADNI participants have been instrumental in two exciting ADNI biomarker studies: (1) providing “gold standard” evidence of LBD and/or MSA pathology for an evaluation of an alpha‐synuclein seeding assay applied to ADNI CSF samples,^34^ and (2) contributing ADNI cohort data to a larger study using MRI to detect non‐AD copathologies.^35^ Additionally, the ADNI NPC is now supporting the mission of CLEAR‐AD (Centrally‐linked longitudinal peripheral biomarkers of AD in multi‐ethnic populations; U19 AG074879) with multiple tissue samples from each of its cases; the multi‐Omic metadata generated by CLEAR‐AD will complement the rich datasets of ADNI and other contributing studies, and foster new biomarker discovery.

As ADNI4 unfolds, the ADNI NPC will continue to expand its cohort of participants registered for brain donation, its database of findings from completed examinations (accessible through LONI), and its collection of highly annotated, thoroughly characterized brain tissue specimens (available through application to the ADNI NP‐RARC and NIA). It will also soon make available and regularly update a comprehensive digital library of its diagnostic histology slides (also available through LONI). As the new ADNI4 cohort – representing more accurately the demographic richness of the US population – matures, the ADNI NPC will also be honored to include its donations as a vital part of the ADNI NPC resource, which is currently very limited in its (self‐reported) ethnic/racial diversity (96% non‐Hispanic White, 3% non‐Hispanic African American, 1% non‐Hispanic Asian in its first 100 completed cases). Such diversification will be essential to broaden the applicability of future ADNI NPC findings. Complementing the rich contributions of other ADNI cores and projects, these offerings from the ADNI NPC will provide vital ammunition in the global fight against ADRD.

## CONFLICT OF INTEREST STATEMENT

J.C.M. is funded by NIH grants P30AG066444, P01AG003991, and P01AG026276. Neither J.C.M. nor his family owns stock or has equity interest (outside of mutual funds or other externally directed accounts) in any pharmaceutical or biotechnology company. R.J.P. is supported by NIH grants R01AG068319, R01 AG053267, R01AG054567, P01 AG003991, P30 AG066444, U19AG024904, U19 AG032438, R01 AG052550, R01 AG070883, R01NS097799, R01NS092865, R01AG054513, and R01 NS075321. Neither R.J.P. nor his family owns stock or has equity interest (outside of mutual funds or other externally directed accounts) in any pharmaceutical or biotechnology company. M.W.W. serves on editorial boards for Alzheimer's & Dementia, MRI, and Topics in Magnetic Resonance Imaging. He has served on advisory boards for Acumen Pharmaceutical, ADNI, Alzheon, Inc., Biogen, Brain Health Registry, Cerecin, Dolby Family Ventures, Eli Lilly, Merck Sharp & Dohme Corp., NIA, Nestle/Nestec, PCORI/PPRN, Roche, University of Southern California (USC), and NervGen. He has provided consulting to Baird Equity Capital, BioClinica, Cerecin, Inc., Cytox, Dolby Family Ventures, Duke University, Eisai, FUJIFILM‐Toyama Chemical (Japan), Garfield Weston, Genentech, Guidepoint Global, Indiana University, Japanese Organization for Medical Device Development, Inc., Medscape, Nestle/Nestec, NIH, Peerview Internal Medicine, Roche, T3D Therapeutics, USC, and Vida Ventures. He has acted as a speaker/lecturer to The Buck Institute for Research on Aging, the China Association for Alzheimer's Disease (CAAD), the Japan Society for Dementia Research, and the Korean Dementia Society. He holds stock options with Alzheon, Inc., Alzeca, and Anven. The following entities have provided funding for academic travel; USC, NervGen, ASFNR, and CTAD Congress. The other authors have no competing interests to declare. Author disclosures are available in the Supporting Information.

## CONSENT STATEMENT

All human subjects provided informed consent for the collection of *ante mortem* data; consent for brain donation was obtained by local legal requirements.

## Supporting information



Supporting information
